# Estimates of Electronic Medical Records in U.S. Emergency Departments

**DOI:** 10.1371/journal.pone.0009274

**Published:** 2010-02-17

**Authors:** Benjamin P. Geisler, Jeremiah D. Schuur, Daniel J. Pallin

**Affiliations:** 1 Institute for Technology Assessment, Harvard Medical School, Boston, Massachusetts, United States of America; 2 Division of Oncology, Harvard Medical School, Boston, Massachusetts, United States of America; 3 Institute of Public Health, Medical Decision Making and Health Technology Assessment, University of Health Sciences, Medical Informatics and Technology, Hall, Austria; 4 Department of Emergency Medicine, Brigham and Women's Hospital, Boston, Massachusetts, United States of America; 5 Department of Medicine, Harvard Medical School, Boston, Massachusetts, United States of America; 6 Division of Emergency Medicine, Children's Hospital Boston, Boston, Massachusetts, United States of America; 7 Department of Pediatrics, Harvard Medical School, Boston, Massachusetts, United States of America; Mount Sinai School of Medicine, United States of America

## Abstract

**Background:**

Policymakers advocate universal electronic medical records (EMRs) and propose incentives for “meaningful use” of EMRs. Though emergency departments (EDs) are particularly sensitive to the benefits and unintended consequences of EMR adoption, surveillance has been limited. We analyze data from a nationally representative sample of US EDs to ascertain the adoption of various EMR functionalities.

**Methodology/Principal Findings:**

We analyzed data from the National Hospital Ambulatory Medical Care Survey, after pooling data from 2005 and 2006, reporting proportions with 95% confidence intervals (95% CI). In addition to reporting adoption of various EMR functionalities, we used logistic regression to ascertain patient and hospital characteristics predicting “meaningful use,” defined as a “basic” system (managing demographic information, computerized provider order entry, and lab and imaging results). We found that 46% (95% CI 39–53%) of US EDs reported having adopted EMRs. Computerized provider order entry was present in 21% (95% CI 16–27%), and only 15% (95% CI 10–20%) had warnings for drug interactions or contraindications. The “basic” definition of “meaningful use” was met by 17% (95% CI 13–21%) of EDs. Rural EDs were substantially less likely to have a “basic” EMR system than urban EDs (odds ratio 0.19, 95% CI 0.06–0.57, p = 0.003), and Midwestern (odds ratio 0.37, 95% CI 0.16–0.84, p = 0.018) and Southern (odds ratio 0.47, 95% CI 0.26–0.84, p = 0.011) EDs were substantially less likely than Northeastern EDs to have a “basic” system.

**Conclusions/Significance:**

EMRs are becoming more prevalent in US EDs, though only a minority use EMRs in a “meaningful” way, no matter how “meaningful” is defined. Rural EDs are less likely to have an EMR than metropolitan EDs, and Midwestern and Southern EDs are less likely to have an EMR than Northeastern EDs. We discuss the nuances of how to define “meaningful use,” and the importance of considering not only adoption, but also full implementation and consequences.

## Introduction

Electronic medical records may improve patient safety and efficiency of care, and universal adoption is a national goal. Physicians and hospitals may receive financial incentives through Medicare and Medicaid if they are “meaningful users” of electronic medical records [1], [2].

The decision to offer incentives reflects the fact that uptake of such systems has been remarkably slow. Recent data found that only 1.5% of US hospitals had a “comprehensive” electronic medical record (EMR), and an additional 7.6% a “basic” system (Jha et al.) [3]. Computerized provider order entry (CPOE) was implemented in only 17%. Only 4% of office physicians had a comprehensive EMR, and 13% a basic one (DesRoches et al.) [4].

EMRs may be particularly important in the emergency department (ED) setting. Care is unscheduled and therefore paper records are unlikely to be retrieved; many patients are unstable and require risky interventions; patients and providers lack a long-term relationship; and patients may be unable to give crucial information due to altered mental status. Nonetheless, studies in the ED setting have also found slow uptake. In 2001–2002, 31% of EDs reported having any form of EMR [5]. A 2000 study of emergency medicine residency-affiliated EDs – the vanguard of the field – found that only 7% of respondents had fully-implemented technology for medication error checking, 18% for CPOE, and 21% for clinical documentation [6]. A 2006 survey of Massachusetts EDs found that only 11% had fully-implemented technology for medication order error checking [7].

Incentivizing healthcare providers to implement EMRs can succeed only if policymakers know what is already in place. We provide this information for the ED setting, using a large, nationally-representative sample of data collected as part of the National Hospital Ambulatory Medical Care Survey (NHAMCS). Our goal is to provide a detailed description of the information technology resources available in the nation's EDs. In reporting our results, not only do we provide objective information about EMR availability in the nation's EDs; we also consider the various ways in which a given ED might be considered to use such technology “meaningfully.” We report the proportion of US EDs that could claim to be “meaningful users” in 2005–2006, under various definitions, including that used by Jha and DesRoches, and consider the validity of various categorization schemes [3], [4]. We expected to find not only that many EDs reported not having adopted an EMR, but that many of those that had could not meet the standard of “meaningful use.” Finally, we sought to determine whether ED or patient characteristics predicted EMR adoption. Overall, our goal is to provide the information policymakers need to understand and influence the implementation of EMRs in the nation's EDs.

## Methods

### Study Design and Sample

NHAMCS is a nationally-representative multi-stage probability survey of visits to all non-institutional, non-Federal hospital EDs, whose methods have been detailed previously [8]. Each year's survey includes two components: the hospital induction survey and chart abstractions. The hospital induction survey includes questions about hospital ED policies and structures, including multiple questions regarding the presence and components of an EMR. ED chart abstractions are performed at each participating hospital and include patient demographic, clinical and administrative data elements. The publicly-available data include weighting variables to allow national estimates of ED-level or patient-level characteristics. We analyzed data from NHAMCS, using the merged datasets from 2005 and 2006, with no exclusions. The institutional review board of Brigham and Women's Hospital has exempted from review all analyses of NHAMCS publicly-available anonymous data.

### Outcome Measures

Our study has two main outcome measures. The first is the proportion of EDs surveyed that reported possessing any EMR. The second is the prevalence of individual EMR functionalities. This is a descriptive outcome, which seeks simply to report the proportions of US EDs reporting use of each of the technologies ascertained by the NHAMCS surveyors.

Secondarily, we explore the concept of “meaningful use,” by applying the definitions of Jha and DesRoches, classifying EMRs as “basic” (including only demographic information, CPOE, lab and imaging results), “basic with clinical notes,” or “comprehensive” (including the above, plus electronic prescribing, radiographic image display, and decision support) [3], [4]. We consider the limitations of this classification scheme, and discuss other ways in which the effort to establish a definition of “meaningful” might be approached.

In order to quantify the extent to which having “any” EMR can misrepresent whether a facility has “meaningful use” of an EMR, we report the rate of adoption of “meaningful” EMRs as a proportion of EDs that had any EMR.

### Statistical Analysis

All analyses accounted for the complex survey design, using recommended sample weights [9], and were performed with SAS 9.1.3 (SAS Institute, Cary, NC). Our main outcomes were descriptive. We report proportions and their 95% confidence intervals (95%CI).

We used multivariate logistic regression to identify predictors of adoption of at least a “basic” system, at the patient level (age, gender, race/ethnicity, source of payment), and at the hospital level (region, metropolitan vs. non-metropolitan [i.e. urban vs. rural], ownership, and teaching status). We first examined the relationship between each predictor variable and the adoption of at least a “basic” EMR using chi-squared statistics. We then constructed multivariate logistic regression models examining the effect of predictor variables on the outcome of interest, adoption of at least a “basic” system. To avoid over-fitting, we included only predictors with univariate p-values ≤0.20 in the model, and optimized it via stepwise backward elimination until all remaining independent covariates had p<0.05 in their type 3 analysis of effects.

## Results

Based on the NHAMCS data, obtained from 694 EDs, 46% (95%CI 39–53%) of US EDs had an EMR in 2005–2006. More than half (59%) of all US ED visits in 2005–2006 were to an ED with an EMR. What it meant to have an EMR varied greatly among these EDs (Table 1). Few (21%) of the nation's EDs had CPOE, and about a quarter (26%) captured and displayed clinical notes electronically. Fewer than half (40%) had at least one decision support functionality, and only one in twenty had all decision support functionalities listed.

**Table 1:** Adoption of Individual Electronic Medical Record Functionalities by US Emergency Departments by 2005–2006.

Category	Subcategory	% (95%CI) of all Emergency departments	% (95%CI) of emergency departments claiming to have any electronic medical record	% (95%CI) of all US emergency department visits
**Historical information**	Patient demographics	43 (36–50)	94 (89–98)	56 (51–62)
	Clinical notes	26 (20–32)	57 (49–65)	35 (30–40)
	Notes including medical history and follow-up†	23 (17–29)	50 (40–60)	33 (27–39)
**Order-entry management**	Orders for medications	21 (16–27)	46 (38–54)	31 (27–36)
	Medication orders sent to pharmacy electronically?†	7 (4–10)	15 (9–22)	11 (6–15)
	Orders for tests	36 (29–42)	78 (71–86)	48 (42–53)
	Test orders sent electronically?†	28 (21–35)	60 (50–71)	38 (32–45)
**Results management**	Viewing laboratory results‡	42 (35–49)	91 (87–95)	54 (48–59)
	Viewing imaging results†	34 (26–42)	73 (64–83)	47 (40–53)
	Electronic images returned†	19 (14–25)	42 (33–52)	30 (23–35)
**Decision support systems**	Drug interaction or contraindication warnings†	15 (10–20)	33 (24–42)	23 (17–30)
	Out-of-range levels highlighted†	31 (23–38)	66 (57–75)	40 (33–46)
	Guideline-based reminders	15 (11–20)	34 (26–41)	21 (18–25)
	*One of the above*	*40 (31–48)*	*86 (80–91)*	*51 (45–57)*
	*All of the above*	*5 (2–7)*	*10 (5–14)*	*7 (4–10)*
**Surveillance**	Public health reporting	12 (7–17)	25 (17–34)	20 (14–25)
	Notifiable disease reporting†	5 (3–7)	10 (6–14)	9 (6–13)
**Any functionality**	Any of the above	46 (37–55)	99 (98–100)	59 (52–66)
**As Classified by Jha and DesRoches** * **[3]**, **[4]**	At least “basic”	17 (13–21)	37 (29–45)	25 (21–30)
	At least “basic with clinical notes”	12 (9–16)	12 (9–16)	17 (14–21)
	“Comprehensive”	6 (3–8)	12 (8–16)	7 (5–9)

† Question only available in 2006 dataset.

‡ Question in 2005 survey asked for all test results, not broken out by lab vs. radiology.

* Jha and DesRoches classified EMRs as: “basic” (including only demographic information, CPOE, lab and imaging results), “basic with clinical notes,” or “comprehensive” (including the above, plus electronic prescribing, radiographic image display, and decision support).

Even among EDs that reported having an EMR, certain key functions were often absent. For example, of EDs reporting that they had an EMR, 94% recorded patient demographics electronically, but only 15% had computerized medication order entry with orders sent to the pharmacy electronically.

With reference to the classification scheme used by Jha et al. [3], only 17% of US EDs met the standard for a “basic” EMR, while 37% of EDs reporting that they had an EMR met this standard. Only 6% of US EDs or 12% of EDs reporting the presence of an EMR met the criteria for a “comprehensive” EMR. Among the 694 participating EDs, none reported having implemented all EMR functionalities.

ED volume was not reported in our source data, but the fact that proportion of patient visits seen in EDs with EMRs (first column of numbers) is larger than the proportion of EDs with EMRs (last column), reveals that EMRs are more prevalent in higher-volume EDs.

As described in Methods, we fit a multivariate model to identify patient and ED characteristics that might predict adoption of at least a “basic” EMR. The final model had geographic region and urban vs. rural as predictors, as no other predictors were significant upon controlled analysis. The analysis revealed that rural EDs were substantially less likely to have at least a “basic” EMR system than urban EDs (odds ratio 0.19, 95%CI 0.06–0.57, p = 0.003), and that Midwestern EDs (odds ratio 0.37, 95%CI 0.16–0.84, p = 0.018) and Southern EDs (odds ratio 0.47, 95%CI 0.26–0.84, p = 0.011) were substantially less likely than Northeastern EDs to have at least a “basic” EMR (Table 2).

**Table 2:** Predictors of Adoption of at least a “Basic” Electronic Medical Record System.*

Category	P-value	Subcategory	Odds ratio (95% confidence interval)
**Metropolitan statistical area status**	0.0034	Urban	reference group
		Rural	0.19 (0.06–0.58)
**Region**	0.0214	Northeast	reference group
		Midwest	0.37 (0.16–0.84)
		South	0.47 (0.26–0.84)
		West	0.93 (0.41–2.12

* In this analysis, we used adoption of a “basic” electronic medical record system as the outcome. This definition was taken from Jha and DesRoches, and required a system to include electronic management of demographic information, computerized provider order entry, and lab and imaging results [3], [4]. We began our analysis by conducing bivariate analyses, to determine which of a series of candidate predictors appeared to have a relationship with the outcome variable. We used the following candidate predictors: patient age, gender, race/ethnicity, and source of payment, and, at the hospital level, region, metropolitan vs. non-metropolitan (i.e. urban vs. rural), ownership, and teaching status. Candidate predictors were eliminated from further consideration if bivariate chi-squared testing resulted in a p-value≥0.20. Remaining candidate predictors were fitted to a multivariate logistic regression model, constructed via stepwise backward elimination until all remaining independent covariates had p<0.05 in their type 3 analyses of effects. Accordingly, the following predictors were eliminated from the model: patient-level variables, age, gender, race/ethnicity, and source of payment (insurance type); and hospital-level variables, ownership, teaching status. Only region and urban/rural status were significant predictors of adoption of at least a “basic” system.

## Discussion

We analyzed data from a large representative sample of US EDs, and found that 46% had implemented electronic medical records (EMRs) in 2005–2006. This compares favorably with results obtained using similar methods for 2001–2002, which estimated that 31% of US EDs had an EMR [5]. This improvement may be encouraging, but it has been pointed out that the presence of “any” EMR does not ensure that the EMR's functionalities are relevant to patient safety or other outcomes. In other words, the mere presence of an EMR does not imply “meaningful use” of health information technology [1].

When we used the classification of Jha and DesRoches, and considered a “basic” system to be the minimum required for “meaningful use,” we found that only 17% of the nation's EDs could claim “meaningful use” [3], [4]. Of EDs reporting that they had an EMR system, fewer than 1/3 had a “meaningful” one according to this definition.

Higher-volume EDs were more likely to report at least a “basic” EMR. This makes sense, because purchase of an EMR system would be much more costly on a per-revenue basis for smaller EDs. It can be argued, moreover, that the unintended consequences of EMR adoption, such as increased waiting times, would not vary according to ED size. Thus, cost and other negatives of EMR adoption would seem to weigh more heavily in the risk-benefit calculation for smaller-volume facilities. To our knowledge, this consideration has not been incorporated into current plans to incentivize EMR adoption on a national basis [2].

We believe that the ED is a particularly important setting for analysis of EMR implementation, and not only because EDs may uniquely benefit from EMRs, due to the urgency of information needs and the lack of long-term relationships between patients and providers. It is also important because EDs may be uniquely vulnerable to the unintended consequences of EMRs. EDs often acquire EMRs but fail to implement them [6]. EMRs and related process-of-care changes are expensive, can *increase* ED length of stay, and can sometimes *impair* patient safety [10], [11], [12].

We chose to analyze the EDs in our data set according to the classification of Jha and DesRoches, since this schema was used in the two largest national surveys of EMR adoption [3], [4]. However, contemplation of these criteria reveals them to be somewhat arbitrary. For example, it is not intuitive that CPOE should be an element of the simplest (“basic”) system, but that display of clinical notes would be an element of a more-advanced system. Our data reveal that many EDs are capable of various electronic functions, but few meet Jha and DesRoches' standards for “basic” or better EMR systems.

Alternative definitions of “meaningful use” could easily be devised. For example, a simple criterion might be implementation of CPOE. This would be attractive not only for its simplicity, but also because CPOE is a prerequisite for checking for errors, cross-reactions, and allergies. Another way to define “meaningful use” might be the ability to display past medical history information, which offers providers critical data about ED patients whom they have never met before. A third definition might rely upon decision support systems. Our data reveal that while the “basic” EMR definition used by Jha [3] and DesRoches [4] would allow only 17% of US EDs to claim “meaningful use,” a definition based on the availability of CPOE, clinical notes, or any decision support, would allow 21%, 26%, or 40% of EDs to make this claim. This demonstrates that varying the definition of “meaningful use” affects the number of facilities that can claim it (and receive financial rewards from the government and other third-party payers).

Fortunately, the US government's operationalization of the concept of “meaningful use” is nuanced and flexible. The definition is still evolving, but is planned to roll out in three stages [2]. In the first stage, eligible providers and hospitals will be graded according to their implementation of roughly 25 functionalities. Stages 2 and 3 would build upon stage 1 functionalities to target specific goals [2]. As mentioned above, we are not aware that any consideration of facility volume has been incorporated into these plans.

The principal limitation of our study is its retrospective nature: we were only able to analyze data that had been collected. Furthermore, we were unable to assess EMR interoperability, i.e. use in more than one institution or department, another important feature of “meaningful” EMR use. The scope of our investigation was limited to surveillance, and we did not attempt to measure the effectiveness or cost-effectiveness of ED EMR systems.

In summary, though the proportion of US EDs reporting EMRs is increasing, fewer than half had such systems in 2005–2006. Among those EDs that did have an EMR, there was a great deal of variation in what the EMR systems could do, and any grading system will reward or penalize the nations EDs quite differently, depending on the chosen definition of “meaningful use.” We end on a cautionary note: more is not always better. In the rush to incentivize implementation of EMR systems, we should study not only the rate of technology acquisition, but also the more-subtle questions of implementation, unintended consequences, and cost-effectiveness. If an ED claims to have a given EMR functionality, is that functionality implemented in full [6]? Is it truly benefitting the ED's clientele, after accounting for all intended and unintended consequences? To answer these questions, how should the government audit EMR adoption and monitor its effects?
